# Rationale and design of the LIBERATES trial: Protocol for a randomised controlled trial of flash glucose monitoring for optimisation of glycaemia in individuals with type 2 diabetes and recent myocardial infarction

**DOI:** 10.1177/1479164120957934

**Published:** 2020-10-20

**Authors:** Colin C Everett, Catherine Reynolds, Catherine Fernandez, Deborah D Stocken, Linda D Sharples, Thozhukat Sathyapalan, Simon Heller, Robert F Storey, Ramzi A Ajjan

**Affiliations:** 1 Clinical Trials Research Unit, University of Leeds, Leeds, West Yorkshire, UK; 2 London School of Hygiene and Tropical Medicine, University of London, Bloomsbury, London, UK; 3 Hull Work Medical School, University of Hull, Hull, East Yorkshire, UK; 4 Department of Oncology and Metabolism, Sheffield Teaching Hospitals Trust, Sheffield, South Yorkshire, UK; 5 Department of Infection, Immunity and Cardiovascular Disease, University of Sheffield, Sheffield, South Yorkshire, UK; 6 Leeds Institute for Cardiovascular and Metabolic Medicine, University of Leeds, Leeds, West Yorkshire, UK; 7 Department of Diabetes and Endocirnology, Leeds Teaching Hospitals Trust, Leeds, West Yorkshire, UK

**Keywords:** Type 2 diabetes mellitus, continuous glucose monitoring, euglycaemia, myocardial infarction, protocol

## Abstract

Hyperglycaemia in individuals with type 2 diabetes (T2D) and myocardial infarction (MI) is associated with guarded clinical prognosis. Studies improving glucose levels in T2D following MI relied on HbA1c as the main glycaemic marker, failing to address potential adverse effects of hypoglycaemia and glucose variability. We describe the design of the LIBERATES trial that investigates the role of flash glucose monitoring in optimising glycaemic markers in high vascular risk individuals with T2D. This multicentre trial is designed to recruit up to 150 insulin and/or sulphonylurea-treated T2D patients, within 5 days of a proven MI. Individuals will be randomised 1:1 into intervention and control groups using flash glucose monitoring sensors and traditional self-monitoring of blood glucose, respectively. The control group will also wear a blinded continuous glucose monitoring sensor. The primary outcome is the difference in time spent in euglycaemia (defined as glucose levels between 3.9–10.0 mmol/l), comparing study groups 3 months following recruitment, assessed daily for 14 days and as an average. Secondary and exploratory end points include time spent in hypoglycaemia and hyperglycaemia, HbA1c, quality of life measures, major adverse cardiac events and cost-effectiveness of the intervention. This study will establish the role of flash glucose monitoring in glycaemic management of individuals with T2D sustaining a cardiac event.

(Trial Registration: ISRCTN14974233, registered 12th June 2017)

## Introduction

Following myocardial infarction (MI), patients with diabetes have worse clinical outcome compared to individuals with normal glucose metabolism, including increased rate of recurrent events and higher cardiovascular and all-cause mortality.^1–4^ This adverse outcome is particularly pronounced in insulin-treated patients.^5^ National and international guidelines recommend aggressive management of dyslipidaemia and hypertension in high vascular risk diabetes patients, though the same guidelines note that a gap in the knowledge exists as to appropriate glycaemic management in this population.^6^

Given the guarded prognosis in those with hyperglycaemia and recent MI,^7–9^ a number of trials have attempted to investigate the effects of reducing glucose levels on clinical outcome.^10^ However, these trials used HbA1c as the sole glycaemic marker, which fails to take into account hypoglycaemia and glucose variability, both of which are associated with adverse clinical outcome.^11–15^ Hypoglycaemia is particularly relevant as intensive glycaemic control results in increased incidence of low glucose levels. A meta-analysis by Boussageon et al.^16^ of randomised controlled trials (not restricted to recent MI) reported a pooled risk of significant hypoglycaemia in patients undergoing intensive glycaemic control to be over two-fold higher than standard control [RR 2.33 99% CI (1.62 to 3.36)]. Moreover, the DIGAMI-2 study^10^ – attempting to optimise glycaemic control in diabetes patients with myocardial infarction – unexpectedly observed a numerically higher mortality rate in intervention groups, particularly in insulin-treated patients, suggesting a detrimental effect of hypoglycaemia when attempting tighter glucose control. This effect was also seen in a systematic review and meta-analysis of 6 cohort studies by Goto et al.^17^ observing a two-fold pooled relative risk of cardiovascular disease among patients with hypoglycaemia [HR: 2.05 (95% CI 1.74 to 2.42)], which was not explained by associated comorbid illnesses.

Glycaemic studies have used HbA1c as the main glycaemic marker given ease of measurement and the difficulties encountered in undertaking and interpreting self-monitoring of blood glucose (SMBG) using capillary testing. The main disadvantage of SMBG is the intermittent nature of testing, providing limited data, and the opportunity for testing arising mainly when the patient presents feeling unwell, thus providing a biased impression of the results. Therefore, the incomplete glycaemic profile provided by SMBG may contribute to the uncertainty surrounding cost-effectiveness of maintaining tight glycaemic control in this clinical situation.^18,19^

While traditional continuous glucose monitoring (CGM) provides more comprehensive glycaemic data, clinical use has been limited due to need for regular calibration, relatively high costs and patient inconvenience due to bulky devices.^20^ However, the recently-introduced Freestyle Libre may address this unmet need by providing a convenient and comprehensive glycaemic review with relatively manageable costs. The Freestyle Libre consists of a factory-calibrated sensor that measures interstitial glucose, placed on the arm with each sensor lasting for 2 weeks. It measures glucose every 15 min and uses an ambulatory glucose profile (AGP) to provide an accurate, comprehensive method for the health care professional to adjust glycaemic therapy safely and effectively.

Previous randomised controlled trials have shown that Freestyle Libre reduces hypoglycaemic exposure both in type 1 and type 2 diabetes patients with the added benefit of reducing HbA1c in inadequately controlled type 2 diabetes (T2D) patients younger than 65 years.^21,22^ Moreover, real-world data in over 50,000 users have shown that device use is associated with high frequency of glucose checks, which demonstrated an inverse correlation with time spent in hyper- or hypoglycaemia.^23^ A more recent study in individuals with T2D has shown that Freestyle Libre use is associated with a significant reduction in HbA1c and improved quality of life measures.^24^ However, it remains unclear whether Freestyle Libre offers benefits in those with associated vascular co-morbidities, particularly following an acute event.

Therefore, we hypothesised that a modern glycaemic monitoring strategy would optimise glucose levels in diabetes patients following MI and improve quality of life in this population. The LIBERATES trial (improving gLucose in patIents with diaBEtes following myocaRdial infArction: The role of a novEl glycaemic monitoring Strategy) has been designed to investigate the role of Freestyle Libre in patients with T2D following acute MI, which we describe in detail including study design, outcome measures and planned statistical analysis.

## Methods

List of abbreviations is provided in Table 1.

**Table 1.** List of abbreviations.

ADDQoL	Audit of Diabetes Dependent Quality of Life
AGP	Ambulatory Glucose Profile
CI	Confidence Interval
CGM	Continuous Glucose Monitoring
CRF	Case Report Form
CTRU	Clinical Trials Research Unit
DTSQs	Diabetes Treatment Satisfaction Questionnaire (status)
EASD	European Association for the Study of Diabetes
EQ-5D-5L	Euroqol 5-dimension health questionnaire
ESC	European Society of Cardiology
HbA1c	Glycated Haemoglobin
HRA	Health Research Authority
MACE	Major Adverse Cardiovascular Events
MI	Myocardial Infarction
NHS	National Health Service
NICE	National Institute for health and Care Excellence
NIHR	National Institute for Health Research
NSTEMI	Non-ST Elevation Myocardial Infarction
PIL	Patient Information Leaflet
PIN	Personal Identification Number
QALY	Quality-Adjusted Life Year
REC	Research Ethics Committee
RfPB	Research for Patient Benefit
RUSAE	Related and Unexpected Serious Adverse Event
SMBG	Self-Monitoring of Blood Glucose
STEMI	ST Elevation Myocardial Infarction
T2D	Type 2 Diabetes

### Trial design

LIBERATES is a multicentre, 2-group, 1:1 randomised, parallel-group, open-label Phase 2 trial investigating the role of flash glucose monitoring, compared with SMBG using capillary glucose testing, for improving glycaemic parameters and quality of life in a high-risk population of patients with T2D who have recently experienced MI.

Recruitment opened in August 2017 and the trial is recruiting from nine UK secondary care hospitals (Leeds, Sheffield, Hull, Nottingham, Birmingham, Morecambe, Wakefield, Reading and Stevenage). The trial is expected to complete follow-up in January 2020.

### Objectives

The primary objective is to estimate the difference in time per day spent in euglycaemia, defined in this trial to be 3.9 to 10.0 mmol/L inclusive, between study groups at 3 months following a cardiac event.

Secondary objectives are to investigate differences in time spent in euglycaemia by 30 days post randomisation, times in hypoglycaemia and hyperglycaemia, HbA1c, and quality of life as well as reporting major cardiovascular events and summarising cost-effectiveness.

### Trial setting

Patients with MI will be recruited during admission to secondary care UK hospitals. Recruitment will primarily be from cardiology wards. All patients will be recruited during their hospital stay and within 5 days of sustaining a MI.

### Eligibility criteria

Patients who satisfy all of the following inclusion criteria, and none of the exclusion criteria are eligible to take part in LIBERATES. Eligibility waivers are not permitted.

#### Inclusion criteria

1. Patient aged 18 years or older;
2. Pre-admission diagnosis of T2D;
3. Pre-admission treatment of hyperglycaemia with a sulphonylurea and/or insulin, with or without additional hypoglycaemic agents;
4. MI defined as typical symptoms of cardiac ischaemia associated with a typical rise in troponin levels using the 99th percentile threshold cut-off (patients with ST-elevation MI and non-ST elevation MI are eligible to take part);
5. Patient has provided informed consent.

#### Exclusion criteria

1. Solely diet-controlled T2D pre-admission;
2. Patient with active malignancy, other than localised squamous cell or basal cell skin carcinoma;
3. Patient known to be pregnant, or requires dialysis;
4. Patient unable to follow study instructions or considered unsuitable for trial participation at the discretion of the treating clinician/nurse;
5. Patient previously participated in the LIBERATES trial.

Patients with existing pacemakers were initially excluded from the trial, but this criterion was relaxed following an amendment to the protocol, approved by the Research Ethics Committee (REC) in April 2019

### Randomisation and blinding

Patients who are eligible and provide written informed consent will be randomly allocated to either flash monitoring of blood glucose (intervention) or standard self-monitoring (control). Random allocation is provided by a central telephone- and internet-based service operated, by the Clinical Trials Research Unit (CTRU) and only accessible by authorised staff, who must log-in with personalised access codes. Allocation sequences (stratified by centre and current use of insulin) using Soares and Wu’s^25^ algorithm are pre-generated for each site by the trial statistician.

Patients and clinicians will not be blind to their randomised allocation. In the standard care group, neither patients nor clinicians are able to see the Libre Pro sensor data nor summary reports arising. In the experimental group, sensor data (and summary reports) will be available to both patients and clinicians to act on as they see fit, either by life-style modifications or by changing hypoglycaemic medications. A simple guide for glycaemic management is provided to study teams but the final decision on treatment changes to be left at the descretion of study investigator at each site (suppl Appendix I). The summary reports available to clinicians in the intervention group include times in euglycaemia, hyper- and hypoglycaemia, so clinicians are not blind to the outcome measures in this group.

### Interventions

#### Experimental – flash monitoring of glucose measurements

Participants randomised to the experimental group will monitor glucose levels by use of the flash glucose monitoring system.

Participants will have a Freestyle Libre sensor (Abbott Diabetes Care, Alameda, CA, USA) applied to the upper arm. The sensor is similar in design to that for the control group.

Should the sensor become detached, the participant will be asked to self-apply a replacement sensor, using the supplied sensor applicator device. Participants will be asked to return all sensors used between days 0-15, 16-30 and 76-91 at the clinic visits. Only data downloaded from the sensors worn between days 0-30 and 76-91 will be transferred to the CTRU for analysis.

The sensor begins recording glucose levels 60 min after activation, automatically records every 15 min, and holds 8 h of data at any one time. Participants will also receive a handheld reader, with which they may scan the sensor to display current glucose levels and download the latest 8 h of data recorded on the sensor. Each individual is required to scan at least three times/day to ensure full 24-h glucose coverage (96 glucose readings/day), though individuals may scan more frequently.

The output provides a record of each observation, comprising date and time of the reading and recorded glucose level in mmol/L. Additional data available include any capillary glucose readings by finger-prick tests performed in addition to the sensor readings – the frequency of which will be reported – as well as medication dosages and relevant dietary information, none of which is anticipated to be used for analysis. Medication changes will be recorded at follow-up visits on case report forms (CRFs) for all participants. The sensors have a recording range of 2.2 mmol/L to 27.8 mmol/L; values outside this range are set to the limit values. Since our outcome measures relate to time above, below or within standard ranges (e.g. <3.9 mmol/L for hypoglycaemia, >10.0 mmol/L for hyperglycaemia), this is not expected to impact on our analyses.

The handheld scanner used to download the sensor readings may also be used to measure capillary glucose levels by means of finger-prick testing (for example, to confirm reports of hypoglycaemia). Patients will be provided with a supply of glucose testing strips and will be asked to use only the intervention reader for such testing and to stop using other glucose metres during the study.

Fourteen day summaries of the time in target range, (3.9–10.0 mmol/L), average glucose (mmol/L), number of episodes and duration (minutes) of low-glucose (<3.9 and <3.0 mmol/l) and sensor use obtained by Freestyle Libre can be used to manage the treatment of participant’s blood glucose levels. The treating clinician may alter the patient’s glucose-lowering therapy at their own discretion and/or according to relevant guidelines based on the reported data.

#### Standard care – self-monitoring of blood glucose

In the standard care group, patients will use capillary glucose testing to self-monitor for 91 days post registration on a regular basis. To ensure standardisation of readings across the two group, standard care participants will be provided with a Freestyle glucose metre and Optium glucose testing strips (Abbott Diabetes Care, Alameda, CA, USA) and instructed to discontinue use of other glucose metres for the duration of the 91-day study period. Participants will use their own lancing device for the purpose of drawing blood for finger-prick testing and taking actions to control their blood glucose levels. Site research nurses will update knowledge on glucose testing and will familiarise patients with the new glucose metre. At follow-up visits, data from the glucose metre will be downloaded and used by the attending team to adjust glycaemic therapy in line with local management policies.

For the purposes of endpoint data collection during the time periods 0-15 days, 16-30 days and 76-91 days, standard care group participants will also have a Freestyle Libre Pro (Abbott Diabetes Care, Alameda, CA, USA) sensor applied to their skin. Freestyle Libre pro sensor is identical to the sensor used in the interventional arm of the study except for having glucose data blinded to patients, health care professionals and study investigators. Should the sensor become detached prior to the end of the recording period, participants will visit their recruiting hospital to have a new sensor attached, to ensure sufficient data records.

The sensor begins recording glucose levels 60 min after activation and automatically records glucose every 15 min (up to 96 readings per day) for up to 14 days. At day 15, 30 and 91 visits, the research team will remove the sensor and download the results using the Freestyle Libre Pro reader. Neither participant nor treating clinician/nurse will have access to this data at any time, so these data will not influence treatment recommendations.

The output data for analysis is in the same format as for the intervention group, with the same recording ranges.

No summary report will be generated for the patients in the standard care group. The treating clinicians will adjust the patient’s glycaemic therapy based on the results of conventional clinical follow-up and any self-monitoring blood glucose results the patient may provide.

### Outcome measures

#### Primary outcome measure

Glucose control, measured by the time per day that glucose is in the range 3.9–10.0 mmol/L inclusive,^26^ measured between days 76–91.

#### Secondary outcome measures

- Glucose control, measured by the time in range (3.9–10.0 mmol/L inclusive) per day, between days 15 and 30;
- Glucose control, measured by the time per day in hyperglycaemia (glucose > 10.0 mmol/L) between days 15 and 30, and days 76 and 91;
- Glucose control, measured by the time per day in hypoglycaemia (glucose < 3.9 mmol/L) between days 15 and 30, and days 76 and 91;
- HbA1c (mmol/mol) value at day 91;
- Weight (Kg) value at day 91;
- Blood Pressure (Systolic and Diastolic) values at day 91;
- Health and treatment-related quality of life measured by EQ-5D-5L utility score, Diabetes Treatment Satisfaction Scale score and Audit of Diabetes Dependent Quality of Life questionnaire domain and overall scores at day 91;
- Safety, measured as incidence of mild, moderate and severe pre-specified trial specific adverse events reported from days 0 to 91;
- Cost-effectiveness;

#### Exploratory outcome measures

- Glucose control, measured by the time per day in “significant hypoglycaemia” (glucose < 3.0 mmol/L) between days 76 and 91;
- Glucose control, measured by the time per day in “extreme hypoglycaemia” (glucose < 2.5 mmol/L) between days 76 and 91;
- Major adverse cardiovascular events (MACE) within maximum 12 months;
- Severe hypoglycaemia (defined as requiring 3rd party assistance with recovery) in months 1 to 3 and in months 4 to 12;
- Diabetes-related hospitalisations within maximum 12 months;
- All-cause mortality within maximum 12 months;
- Changes in care, measured by changes in diabetes and other cardiac medication usage within 91 days.

The MACE endpoint is defined as death due to cardiac cause or hospitalisation for one of the following: acute coronary syndrome (including MI and unstable angina); heart failure; unscheduled revascularisation (by percutaneous coronary intervention or coronary artery bypass graft); arrhythmia; cerebrovascular event (including thrombotic or haemorrhagic stroke and transient ischaemic attack).

### Data collection

**Figure 1.**
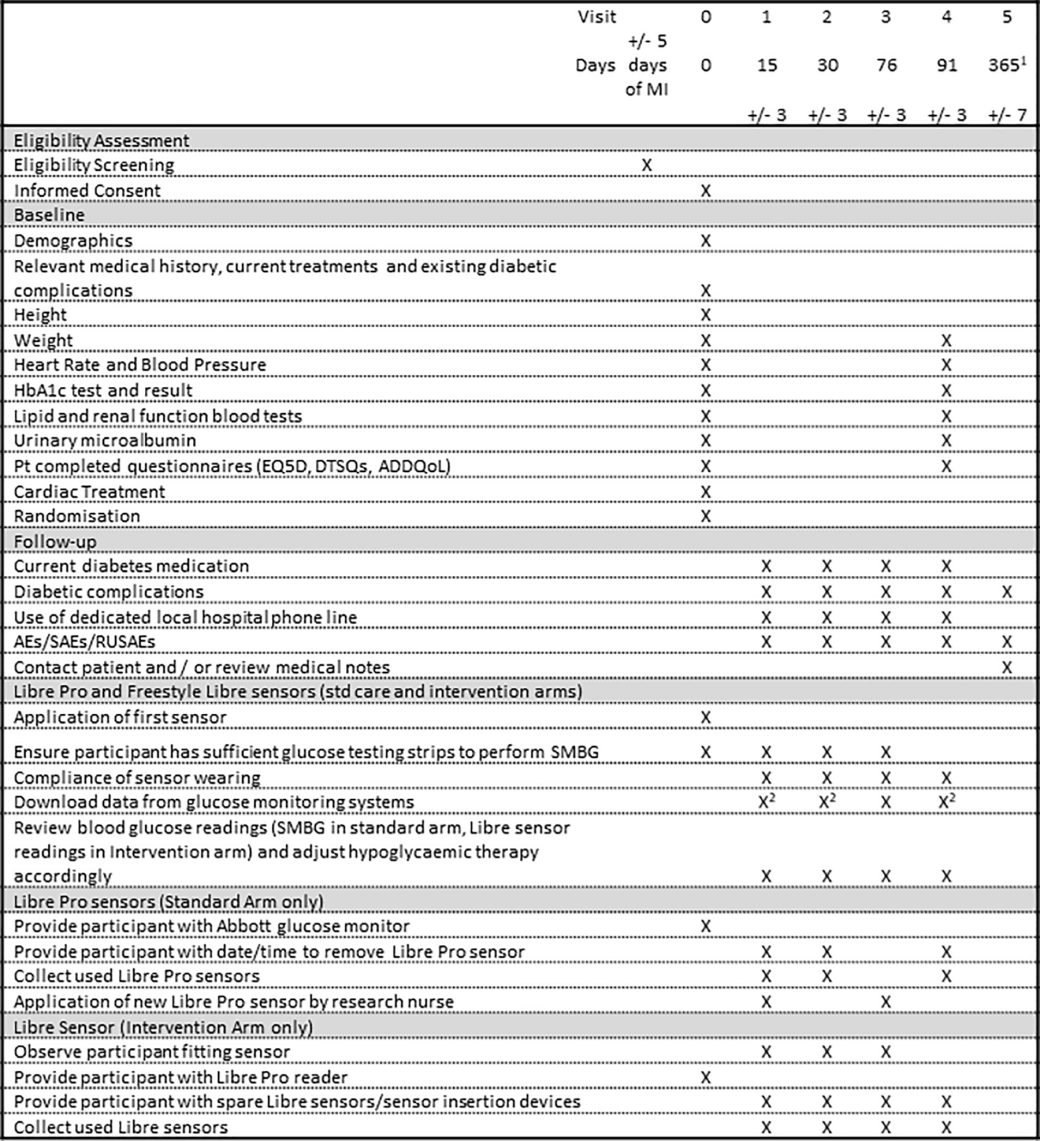
Summary of assessments, and timing of outcome assessments. ^1^The final follow-up visit will take place 12 months post randomisation for patients randomised before 31st January 2019, and at visit closest to 31st January 2020 for those randomised later. ^2^Libre Pro sensor data to be downloaded after the participant has left the clinic and must NOT be viewed by the treating clinician/nurse. SMBG: self-monitoring of blood glucose.

Figure 1 illustrates the data collected at the specific time points in the LIBERATES study.

Clinical data will be recorded at sites on paper CRFs, copies of which will be retained at site while originals will be sent to the CTRU. To ensure patient confidentiality, CRFs will only record the sequential patient trial identifier, and the patient date of birth and initials for verification. Consent forms will record NHS Number and full name and contact details for each patient. Data will be entered onto a database (MACRO, Infermed), which will have specified a number of validation checks to automatically identify certain erroneous data items. Reported discrepancies will be returned to sites for data clarification.

Endpoint data relating to glucose control automatically collected by the glucose sensors will be downloaded at the hospital. Raw glucose levels at each date/time point will be transferred to the CTRU by an encrypted file transfer service and stored electronically in a location accessible only to the trial data manager and statistician. Transferred data files will identify the patient only by referring to the unique trial ID, patient initials, and date of birth, and will identify the study visit number and visit date.

At the day 15, 30, 76 and 91 follow-up visits, participants will be prompted to report the occurrence and frequency of any expected safety events relating to diabetes or to the glucose sensor. Any related and unexpected serious adverse events (RUSAEs) will be reported separately and are subject to expedited review: RUSAEs will be reported to the CTRU within 24 h of the site becoming aware of the event. The event will be reviewed by the Chief Investigator (CI) and will be reported to the sponsor within 1 day of CTRU becoming aware, and reported by the CTRU to the main Research Ethics Committee within 15 days.

Patients recruited prior to 31st January 2019 will have their final follow-up visit 1 year after randomisation. Those recruited between 1st January and end of recruitment will have their final follow-up visit as close as possible to 31st January 2020. At the final follow-up time point, the recruiting hospital will contact the patient (and/or look at the patient’s medical notes) to obtain information on diabetic complications, severe hypoglycaemia, mortality, diabetes-related hospitalisations, and hospitalisation for acute coronary syndrome (including MI and unstable angina), heart failure, unscheduled revascularisation, arrhythmia or cerebrovascular events.

Participants who cease using the sensors will be followed up according to the trial schedule. Participants will have the option to withdraw from the follow-up period for any reason and will have the option to permit staff to access medical records to identify long-term events.

### Statistical considerations

#### Sample size

The sample size of 150 patients is based on a realistic recruitment target and according to a Bayesian decision rule for the primary outcome. Our decision rule would be that progression to an international definitive trial is indicated if the observed posterior probability of a positive treatment effect is 80% or greater. A simulation-based approach was used to estimate posterior probability of a positive difference in time in euglycaemia (in favour of the Freestyle Libre). The following assumptions were made, based on previous published data,^22^ as well as unpublished data from 14 patients each providing measurements of time in euglycaemia for 7 days:

- mean time per day in euglycaemia of 13.3 h in the control group;
- mean time per day in euglycaemia of 14.8 h in the intervention group (effect of 1.5 h);
- between-patient variance of 32.90;
- residual (within-patient) variance of 10.97;
- intra-class correlation of 0.75;
- minimum of 9 days complete data per patient, with days 10-14 each having an independent 20% chance of being unobserved.

Based on 1000 simulations, the expected posterior probability of the difference in the time in euglycaemia (defined in study participants as glucose levels between 3.9-10.0 mmol/l) between the groups being greater than zero (conditional on 1.5 h being both the observed and true difference) would be 0.958 (90% interval 0.946–0.97). In the same scenario, if 20% of participants are lost to follow-up, then the expected posterior probability would be 0.939 (0.921, 0.957). The posterior probabilities calculated assumed an uninformative prior distribution. Table 2 summarises the expected standard errors from the simulations, and the mean (90% percentile interval) of the posterior probabilities for a range of sample sizes and potential observed intervention effects. In summary, with 150 patients, there is a high chance (>90%) of concluding a treatment benefit if the true difference between the groups is at least 1.5 h.

**Table 2.** Results of simulations to estimate standard errors from primary analysis models and expected posterior probabilities of concluding a positive treatment effect. Mean probabilities are given with 90% intervals for the posterior probabilities observed in the simulations. *d*^= observed (and true) treatment effect.

Total sample size (*n* per group)	Expected standard error	Pr (d>0|data)* if $\hat{d}$ =1.5	Pr (d>0|data) if $\hat{d}$ =1.0	Pr (d>0|data) if $\hat{d}$ =0.5
60 (30)	1.353	0.867 (0.837, 0.897)	0.771 (0.744, 0.8)	0.645 (0.628, 0.663)
80 (40)	1.175	0.899 (0.874, 0.924)	0.803 (0.777, 0.831)	0.665 (0.649, 0.684)
100 (50)	1.057	0.922 (0.903, 0.942)	0.828 (0.807, 0.853)	0.682 (0.667, 0.7)
120 (60)	0.967	0.939 (0.921, 0.957)	0.85 (0.827, 0.874)	0.698 (0.681, 0.717)
140 (70)	0.896	0.953 (0.938, 0.967)	0.868 (0.848, 0.89)	0.712 (0.696, 0.73)
150 (75)	0.867	0.958 (0.946, 0.97)	0.876 (0.858, 0.895)	0.718 (0.704, 0.735)

#### Analysis methods

Prior to final analysis, a full Statistical Analysis Plan will define analysis populations, outcome measure derivations, procedures for imputing missing data (and/or conducting analysis in the presence of missing data), and specific analyses for each endpoint, including covariate adjustments, sensitivity analyses and any further exploratory analyses. The CI will approve the final version.

Primary analyses will be on an intention-to-treat (ITT) basis, in which all patients are included in analyses in the group to which they are assigned at randomisation, regardless of study completion or protocol adherence. Sensitivity analyses will consider a per-protocol (PP) analysis (for which a minimum degree of compliance will be agreed by the Trial Management Group) and an appropriate randomisation-respecting efficacy analysis (for example, Principal Stratification).

The primary outcome measure will be analysed and interpreted within the Bayesian framework, estimating and reporting the posterior probability of a positive effect. If this posterior probability is 0.8 or more, then this would indicate that moving to a definitive trial is recommended. The primary analysis will use proper but uninformative prior distributions for all parameters in the model. Sensitivity analysis will consider more informative priors, based on previous published data, as sensitivity analyses.^27,28^ The outcome measure itself will be the time in each individual 24-h period that the patient’s glucose spends in the range [3.9, 10.0 mmol/L], thus each patient will have multiple readings within the 76 to 91 day range. These will be analysed using a hierarchical longitudinal repeated measures model, comprising random intercepts for patients and centres, and random slopes for time, as well as fixed effects for intervention arm, insulin use, time, time-by- intervention interaction and day 0 time in range. We will initially assume a linear effect of time, but alternative patterns of change will be considered when data are available. The Bayesian posterior probability of positive intervention effect will be reported, when using prior distributions for main effects and covariances that are proper and non-informative. An additional analysis will account for possible moderating effect of baseline insulin use on the outcome measure, by including a fixed effect for the interaction between baseline insulin use and the intervention arm. Finally, for an ancillary analysis, we will obtain a single mean average time per day in normal range for each patient, and fit a conventional linear regression model, adjusting for fixed effects of insulin use, mean baseline glucose level and random intercept effects for randomising centre. This analysis will allow comparison between our results and other published trials using such analysis approaches.

Table 3 lists the planned analysis approach for the secondary and exploratory outcome measures. In general, analyses will take the form of regression models that adjust for the stratification factors, reported as estimated effect sizes with 95% confidence intervals. Analyses of “time per day” with glucose in a given range will be derived and analysed analogously to the primary outcome measure, though not within the Bayesian framework.

**Table 3.** Summary of planned statistical analysis approaches for the secondary and exploratory outcome measures.

Outcome measure	Brief analysis plan
Secondary	
Time per day in euglycaemia [3.9, 10.0 mmol/L] (at days 16–30)	Multi-level longitudinal mixed effects linear regression model, adjusted for random centre intercept effects*, fixed effects of insulin use, baseline glucose, time, time-by-treatment interaction and day 0 time in euglycaemia, random effects for patient and patient by time.
Time per day in hyperglycaemia (>10.0 mmol/L) (at days 76–91 and days 16–30)	
Time per day in hypoglycaemia <3.9 mmol/L) (at days 76–91 and days 16–30)	Ancillary analysis: linear regression model, adjusted for fixed/random centre effects*, fixed effects of insulin use and baseline glucose level.
HbA1c	Linear regression, adjusted for fixed / random centre effects*, fixed effects of insulin use and baseline value.
Weight	
Blood Pressure	
DTSQs scores	
EQ5D-5L utility score	
ADDQol scores	
(all at day 91)	
Exploratory	
Time per day in significant hypoglycaemia [<3.0 mmol/L) (at days 76–91 and days 16–30)	Multi-level linear regression model, adjusted for random centre effects*, fixed effects of insulin use, baseline glucose, time, time-by-treatment interaction, random effects for patient and patient by time.
Time per day in extreme hyperglycaemia [< 2.5 mmol/L) (at days 76–91 and days 16–30)	
	Ancillary analysis: linear regression model, adjusted for fixed / random centre effects*, fixed effects of insulin use and baseline glucose level.
Proportions of patients	For all except deaths: Poisson regression, including offset term for duration of follow-up, adjusted for fixed effects of insulin use and random centre effects. Unadjusted differences in proportions between groups.
+ experiencing severe hypoglycaemia that requires third party assistance (in months 1–3 and 4–12) + experiencing MACE within	
maximum 12 months	
hospitalised for diabetes-related causes within maximum 12 months	
+ deceased within maximum 12 months (all causes)	For deaths: Summary statistics by centre, insulin status and group. Unadjusted differences in proportions deceased in the two groups.
Time until first	Kaplan-Meier estimates of proportions event-free, stratified (by centre and insulin use), log-rank test. Time-to-event regression model (e.g. Cox proportional hazards, accelerated failure) adjusted for fixed effect of insulin use and random centre effects*.
+ MACE	
+ Diabetes-related hospitalisation	
+ Death (all causes)	
Use of diabetic and other cardiovascular medications	Summary statistics by group
Adverse Events	

* Centre will be fitted as a random (intercept) effect in the first instance. If centre sizes are too small to permit this and/or result in non-positive variance estimates, we will not adjust for randomising centre. Small centres will not be combined to form larger “pseudo-centres” to improve model fit.

To allow for the possibility of sensor data not being recorded or not available, missing data will be primarily dealt with under the “missing at random” assumption.^29^ Analyses will either use multiple imputation to produce a number of completed datasets – analysed individually before combining resulting estimates – or will use maximum likelihood analysis of mixed models with random patient effects to meet this assumption. Sensitivity analyses will consider the effect of departures from the “missing at random” assumption.

Analyses of secondary outcome measures that may be affected by the 1-year follow-up being truncated will either be assessed by appropriate time-to-event analysis methods or by regression methods including offset terms for the duration of follow-up.

#### Economic evaluation methods

The Cost-Effectiveness analysis will be performed according to the reference case guidance for technology appraisals set out by NICE.^30^

At 91 days, a cost-consequences analysis will be performed in order to contrast the difference in health care cost with the difference in EQ-5D-5L scores (converted to Quality-Adjusted Life Years (QALYs)), diabetes treatment satisfaction levels (measured using DTSQs) and time spent in euglycaemia between the intervention and standard care and control groups.

## Trial governance and oversight

Ethical approval was obtained from the Yorkshire and Humber Leeds East REC on 28th June 2017. (REC reference 17/YH/0163) Approval from the Health Research Authority (HRA) was received on 4th July 2017. The most recent amendment to the protocol was approved in April 2019.

The sponsor for the trial is the University of Leeds. Given the low risk of the intervention, oversight of the study is provided by a single Trial Safety and Oversight committee (TSOC) combining the roles of a Data Monitoring and Ethics Committee and a Trial Steering Committee. The TSOC comprises an independent statistician, two independent clinicians and an independent patient representative. No formal interim analyses of safety or efficacy will be performed, hence there are no rules or guidelines for early trial termination.

After completion of final analysis, the final trial dataset will be property of the trial team and held at CTRU, University of Leeds. The trial team has agreed for a copy of the final clinical study report to be shared with funders (Abbott and NIHR) as part of the contractual agreement, in addition to access to manuscripts and abstracts for publication or dissemination.

No release of trial data will take place until after publication of the main trial results. Access to the trial dataset for bona fide research purposes will require a signed legally-binding contract to ensure the data security and confidentiality of participants and defining particularly the data items requested and planned analyses. Participants will only be included in shared datasets if they consented. Data will be anonymised prior to any data transfer.

## Discussion

We describe the scientific rationale and design of the LIBERATES trial. This trial will recruit T2D patients with recent MI to test whether the use of Freestyle Libre leads to better glycaemic control compared with standard care consisting of self-monitoring of capillary glucose measurements. This trial aims to establish differences in glucose parameters between two study groups in a relatively limited number of patients, akin to other CGM studies. Data from the work will establish the feasibility of a larger scale study investigating hard clinical outcomes in individuals with T2D and recent MI, including MACE and mortality.

Glycated haemoglobin is a convenient outcome measure of average plasma glucose levels over a 3-month period. However, it does not provide an assessment of hypoglycaemia, nor variability in glucose levels, both of which are associated with adverse outcomes. CGM permits such swings to be detected, providing a more comprehensive assessment of glycaemia.

Previous randomised controlled trials with Freestyle Libre in T2D are limited to two studies. The REPLACE study (NCT02082184^22^) recruited 224 inadequately controlled, insulin-treated T2D patients, having HbA1c between 7.5% and 12% (58–108 mmol/mol). Individuals were randomised on 2:1 basis to intervention using flash glucose monitoring and standard control group employing SMBG for glucose measurements. Flash glucose monitoring did not show improvement in HbA1c, although a pre-specified subgroup analysis of patients younger than 65 years showed significant reduction in HbA1c by 5 mmol/mol in the Libre group compared with standard care (after adjustment for baseline). A more recent study on 101 insulin-treated individuals with T2D has shown a significant reduction in HbA1c and improvement in quality of life measures in the group managed with Freestyle Libre compared with SMBG.^24^

The LIBERATES trial is novel in that it targets a higher vascular risk population with T2D patients. This is a particularly difficult group due to patient concerns following a life-threatening event, the need to undergo various procedures and start of new therapies. Therefore, glycaemia often becomes of secondary importance despite the documented association between high glucose levels and adverse clinical outcome.

The planned analysis of LIBERATES is also novel in that continuous glucose traces will not be averaged to create a single value per patient: rather, the trace will be partitioned into individual daily readings, to be analysed as a repeated measures longitudinal analysis. By doing so, the information in the data can be maximised with changes in glucose control modelled over time, and the correlation between measurements made on the same patient taken into account. However, traditional analysis of the aggregated data will also be undertaken to enable comparison with published results from RCTs and observational studies.

The LIBERATES trial will provide valuable information on the use of Freestyle Libre in T2D patients with recent MI who are at risk of hypoglycaemia. It will clarify the role of Freestyle Libre in improving glycaemic parameters and quality of life in this highly stressed group. In particular, multiple glycaemic markers will be studied, thus providing a comprehensive assessment of glycaemia to aid the clinical management of these patients. Also, it will provide preliminary data for a large multinational clinical outcome study investigating the use of this device to improve short and medium-term clinical outcome in diabetes patients post MI.

## Supplemental Material

LIBERATES_Protocol_Publication_Supp_Appendix_V2_0_Submitted – Supplemental material for Rationale and design of the LIBERATES trial: Protocol for a randomised controlled trial of flash glucose monitoring for optimisation of glycaemia in individuals with type 2 diabetes and recent myocardial infarction Click here for additional data file. Supplemental material, LIBERATES_Protocol_Publication_Supp_Appendix_V2_0_Submitted for Rationale and design of the LIBERATES trial: Protocol for a randomised controlled trial of flash glucose monitoring for optimisation of glycaemia in individuals with type 2 diabetes and recent myocardial infarction by Colin C Everett, Catherine Reynolds, Catherine Fernandez, Deborah D Stocken, Linda D Sharples, Thozhukat Sathyapalan, Simon Heller, Robert F Storey and Ramzi A Ajjan in Diabetes & Vascular Disease Research
