# Supplementary material for: Rationale and design of the LIBERATES trial: Protocol for a randomised controlled trial of flash glucose monitoring for optimisation of glycaemia in individuals with type 2 diabetes and recent myocardial infarction
Source: Diab Vasc Dis Res. 2020 Oct 20;17(5):1479164120957934. doi: 10.1177/1479164120957934 (PMC7919208; doi:10.1177/1479164120957934)
Supplement: LIBERATES_Protocol_Publication_Supp_Appendix_V2_0_Submitted – Supplemental material for Rationale and design of the LIBERATES trial: Protocol for a randomised controlled trial of flash glucose monitoring for optimisation of glycaemia in individuals with type 2 diabetes and recent myocardial infarcti [file LIBERATES_Protocol_Publication_Supp_Appendix_V2_0_Submitted.pdf]

Research Article: Trial Design

Rationale and design of the LIBERATES trial: protocol for a randomised controlled trial of flash glucose monitoring for optimisation of glycaemia in individuals with type 2 diabetes and recent myocardial infarction

Colin C. Everett[1], Catherine Reynolds[1], Catherine Fernandez[1], Deborah D. Stocken[1], Linda D. Sharples[2], Thozhukat Sathyapalan [3], Simon Heller[4], Robert F. Storey[5] and Ramzi A. Ajjan[6]

Supplementary Material

Appendix A: WHO Trial Registration Dataset

Appendix B: Non-binding clinician guidance for treatment modification

Affiliations:

[1] Clinical Trials Research Unit, University of Leeds, Leeds, West Yorkshire, United Kingdom.
[2] London School of Hygiene and Tropical Medicine, London, United Kingdom.
[3] University of Hull, Hull, East Yorkshire, United Kingdom.
[4] Sheffield Teaching Hospitals Trust, Sheffield, South Yorkshire, United Kingdom.
[5] University of Sheffield, Sheffield, South Yorkshire, United Kingdom.
[6] Leeds Institute for Genetics, Health and Therapeutics. University of Leeds, Leeds, West Yorkshire, United Kingdom.

Appendix A: WHO Trial Registration Data Set

Trial Design

Parameter	
Primary Registry and Registration ID	ISRCTN14974233
Date of registration in Primary Registry	12th June 2017
Secondary Identifying Numbers	PB-PG-0815-20011
Sources of Monetary / Material Support	NIHR RfPB, Abbot Laboratories
Primary Sponsor	University of Leeds
Secondary Sponsors	None
Contact for Public Queries	Catherine Reynolds, CTRU, University of Leeds, LS2 9JT, United Kingdom.
Contact for Scientific Queries	Dr Ramzi Ajjan, LIGHT, University of Leeds, LS2 9JT. United Kingdom.
Public Title	LIBERATES
Scientific Title	Improving glucose control in patients with diabetes following myocardial infarction: The role of a novel glycaemia monitoring strategy
Countries of Recruitment	United Kingdom
Health Conditions studied	Diabetes Type II, recent-onset myocardial infarction
Intervention(s)	Intervention: Flash-continuous glucose monitoring (Freestyle Libre, Abbot laboratories)<br>Standard: Self-monitoring of blood glucose levels
Key Inclusion and Exclusion criteria	Aged 18 or above; Type 2 diabetes Mellitus (not controlled by diet alone), recent-onset myocardial infarction, use of sulphonylurea and/or insulin (with or without other hypoglycaemic agents) for treatment of hypoglycaemia prior to admission; written informed consent; no active malignancy; neither pregnant nor on dialysis; not previously participated in LIBERATES

Study Type	Interventional,<br>Allocation: randomized;<br>Intervention model: parallel-group.<br>Masking: unblinded;<br>Phase IIb
Date of first enrolment	23 August 2017
Sample Size	Planned: 150. Current 141.
Recruitment status	Recruitment Completed.
Primary Outcome	Time per day in euglycaemia at days 79-91
Key Secondary Outcomes	Time per day in euglycaemia at days 15-30.<br>Time per day in hyperglycaemia (> 10.0mmol/L) at days 76-91 and 15-30<br>Time per day in hypoglycaemia (< 3.9mmol/L) at days 76-91 and 15-30.

Table A1: WHO trial registration dataset.

APPENDIX B – Non-binding treatment guidance for clinician modification

DIABETES TREATMENT INFORMATION GUIDANCE

For participants in both the Standard Arm and the Intervention Arm, the following information is for guidance only, treatment decisions are at the discretion of the treating clinician.

Insulin users

For patients on insulin with or without oral hypoglycaemic agents, assessment will follow the same approach as above and using similar criteria for HbA1c, fasting glucose, post-prandial glucose and hypoglycaemia. Targets may need to be individualised to offer a balance between good glycaemic control and risk of hypoglycaemia. This will occur at the discretion of the investigator after careful review of AGP as outlined in Table 1 below.

Insulin regimen	Fasting glucose > 7.0 mmol/L (x3/week or more)	PP glucose > 10.0 mmol/L (x3/week or more)	Hypoglycaemia < 3.9 mmol/L (x2/week or more)
Basal Only	Increase basal by 5-10% (min 2 and max 8 units)	Introduce short-acting prandial insulin with meals	Reduce insulin by 10-20% (2-8 units)**
Mixed	Increase evening insulin by 5-10% (min 2 and max 8 units)*	Increase pre-meal insulin by 5-10% (min 2 and max 8 units)*	Reduce insulin doses by 10-20%**
Basal bolus	Increase basal by 5-10% (min 2 and max 8 units)	Increase bolus by 5-10% (min 2 and max 8 units)	Fasting or O/N: reduce basal by 10-20%**<br>Postprandial: reduce bolus by 10-20%**

Table 1: Change in insulin doses according to glucose readings. O/N: Overnight; PP: Post-prandial; * consideration can be given to a different mixture of insulin; ** more aggressive reduction in insulin is permitted in cases of severe hypoglycaemia (after discussion with the PI).

Non-insulin users

For patients on sulphonylurea therapy with/without other hypoglycaemia agents, assessment will be based on HbA1c, fasting glucose, postprandial glucose and hypoglycaemia, as follows:

* Increase dose or add in an additional hypoglycaemic agent if <u>any</u> of the following is observed:

i) HbA1c >58 mmol/mol,
ii) Fasting glucose consistently >7.0 mmol/L (≥3 readings/week)
iii) 2 hour postprandial glucose consistently >10.0 mmol/L (>3 readings/ week).

* Patients with significant hypoglycaemia (<3.0 mmol/L) or persistent mild hypoglycaemia (<3.9 mmol/L) will require a reduction in sulphonylurea dose or stopping this therapy.

A switch to insulin therapy can occur in those on maximal oral hypoglycaemic agents with/without glucagon-like peptide (GLP)-1 analogue therapy (escalation of hypoglycaemic therapy to follow NICE guidelines).
